# Complete genome sequence of *Acidovorax temperans* strain LMJ isolated from a contaminated *Chlamydomonas reinhardtii* Tris-Acetate-Phosphate medium culture plate

**DOI:** 10.1128/mra.01293-23

**Published:** 2024-03-15

**Authors:** Mautusi Mitra, Ana Stanescu

**Affiliations:** 1 Department of Natural Sciences, University of West Georgia, Carrollton, Georgia, USA; 2 Department of Computing and Mathematics, University of West Georgia, Carrollton, Georgia, USA; Loyola University Chicago, Chicago, Illinois, USA

**Keywords:** *Acidovorax temperans*, complete genome, xenobiotic tolerance, heavy metal tolerance

## Abstract

A heavy metal and xenobiotic-tolerant strain of *Acidovorax temperans* strain LMJ was isolated from a contaminated Tris-Acetate-Phosphate medium plate of a green micro-alga, *Chlamydomonas reinhardtii*. Here, we report the complete genome sequence of this strain to provide insights into its survival strategies and nearest taxonomic neighbor.

## ANNOUNCEMENT

*Acidovorax temperans* strain LMJ was isolated at our research lab from a contaminated Tris-Acetate-Phosphate medium plate of *Chlamydomonas reinhardtii* strain, Chlamydomonas Library Project LMJ.SG0182, obtained from the Chlamydomonas Resource Center as part of a Carrollton high school student science project (1). The 16S rRNA gene was partially amplified and sequenced to identify the genus as *Acidovorax* sp. [MN633292.1 (1)] for the science project. When we discovered that the strain is heavy metal and xenobiotic tolerant (1), we decided to sequence the genome to provide insight into its environmental adaptation strategies (1).

Genomic DNA was isolated from the cells grown at 23°C aerobically on an LB-agar plate using the protocol in Qiagen’s blood and cell culture DNA mini kit handbook without modifications. A genomic DNA sample was shipped to Georgia Genomics and Bioinformatics Core (GGBC) at the University of Georgia (USA) for PacBio Single Molecule Real Time (SMRT) sequencing. The size range of the fragments post-shearing was determined with a Bioanalyzer 12000 chip, and DNA quantification was performed on a Nanodrop system. Purification and concentration of 12 kb fragment sizes, SMRTbell Library preparation, and its quality assessment and barcoding were performed by GGBC following the protocol stated in the PacBio SMRTbell Library preparation handbook. The barcoded-SMRT bell library was sequenced with two additional barcoded microbial SMRT Bell sequencing libraries in a single SMRT cell using PacBio SMRT Continuous Long Read sequencing on a PacBio Sequel II system, using the PacBio sequencing primer v.4. PacBio sequencing yielded a total of 1,611,871 subreads and a total of 1,514,808 mapped subreads. The mapped subread length *N*_50_ is 10,039 bp. Reads were assembled and circularized using the SMRT Link version 9 software tool (has built-in HGAP v4:0) powered by the PacBio Cromwell workflow engine. The pipeline was run at default with a pre-specified, approximately estimated genome size of 5.3 Mb (based on available complete genome sizes of various *Acidovorax* sp. on NCBI). The genome was also assembled with FLYE v2.9.1 for reliability. We used CANU v2.2 to generate corrected reads. The corrected and trimmed reads and the assembly files were fed into Circlator [v1.5.5 (2)] to identify and trim the overlap to circularize the assemblies. Local assemblies in Circlator were generated using built-in SPAdes (v3.7.1) with a kmer length of 97 bp. The DnaA gene sequence of *Acidovorax* sp. 1608163 (GenBank accession number: CP033069.1) was used to rotate the genome using built-in MUMmer (v3.23) in the Circlator. Contig 1 *N*_50_ and *L*_50_ were 4.5 Mb and 1, respectively (Table 1).

**TABLE 1:** Contig assembly information

Contig and GenBank accession no.	Length (bp)	Circularity	G + C content (%)	Coverage (×)
Contig1/chromosome CP117193.1	4,510,507	Yes	63.5	2,502
Contig 2/plasmid pEP1 CP117194.1	187,379	Yes	60	3,304
Contig 3/plasmid pJG1 CP117195.1	32,883	Yes	62	2,656

The assembled genome was deposited in GenBank (GenBank accession number: GCA_028596105.1). The complete genome contains 4,730,769 bp in three contigs with an average G + C% of 61.83%, where contig 1 is the circularized chromosome, and contigs 2 and 3 are putative plasmids (Table 1). The mean genome coverage is 2,535.7×. The genome was annotated using the NCBI Prokaryotic Genome Annotation Pipeline (PGAP) [v 6.6 revision 2023-10-03.build7061; https://doi.org/10.6084/m9.figshare.25268392.v2; (3, 4)]. The genome quality was assessed using NCBI’s CheckM analysis tool (v1.2.2), which showed completeness of 98.72% and contamination of 0.45% (5). The genome was reannotated using RASTtk v1.073 in the DOE Systems Biology Knowledgebase (KBase) with default parameters (https://kbase.us/n/161632/357/ ) (6–8). The strain genus was identified as *Acidovorax* using GTDB-Tk-v1.7.0 in KBase with default parameters (8, 9). PGAP’s built-in average nucleotide identity (ANI) tool identified the strain as *Acidovorax temperans* strain DSM 7270 (GenBank accession number: GCA_006716905.1), with an ANI of 96.43% (4, 10).

## Data Availability

The whole-genome sequencing project (including the PacBio DNA methylation motifs) has been deposited at GenBank under the accession number GCA_028596105.1. The raw sequence reads have been deposited in the SRA under the accession number SRR23501622. KBase analysis is publicly available in the KBase static narrative (https://doi.org/10.25982/161632.357/2228677). The PacBio DNA methylation motifs have been submitted to REBASE (Ref#35996).
